# Immunobiology of Gestational Diabetes Mellitus in Post-Medawar Era

**DOI:** 10.3389/fimmu.2021.758267

**Published:** 2022-01-03

**Authors:** Surendra Sharma, Sayani Banerjee, Paula M. Krueger, Sandra M. Blois

**Affiliations:** ^1^ Department of Pediatrics, Women and Infants Hospital-Warren Alpert Medical School of Brown University, Providence, RI, United States; ^2^ Department of Obstetrics and Fetal Medicine, University Medical Center Hamburg-Eppendorf, Hamburg, Germany

**Keywords:** gestational diabetes mellitus, regulatory T and Th17 cells, proteinopathy, galectins, animal models

## Abstract

Although the concepts related to fetal immune tolerance proposed by Sir Peter Medawar in the 1950s have not withstood the test of time, they revolutionized our current understanding of the immunity at the maternal-fetal interface. An important extension of the original Medawar paradigm is the investigation into the underlying mechanisms for adverse pregnancy outcomes, including recurrent spontaneous abortion, preterm birth, preeclampsia and gestational diabetes mellitus (GDM). Although a common pregnancy complication with systemic symptoms, GDM still lacks understanding of immunological perturbations associated with the pathological processes, particularly at the maternal-fetal interface. GDM has been characterized by low grade systemic inflammation that exacerbates maternal immune responses. In this regard, GDM may also entail mild autoimmune pathology by dysregulating circulating and uterine regulatory T cells (Tregs). The aim of this review article is to focus on maternal-fetal immunological tolerance phenomenon and discuss how local or systemic inflammation has been programmed in GDM. Specifically, this review addresses the following questions: Does the inflammatory or exhausted Treg population affecting the Th17:Treg ratio lead to the propensity of a pro-inflammatory environment? Do glycans and glycan-binding proteins (mainly galectins) contribute to the biology of immune responses in GDM? Our understanding of these important questions is still elementary as there are no well-defined animal models that mimic all the features of GDM or can be used to better understand the mechanistic underpinnings associated with this common pregnancy complication. In this review, we will leverage our preliminary studies and the literature to provide a conceptualized discussion on the immunobiology of GDM.

## Introduction

Obesity, diabetes, insulin treatment, stress, and hypertension are words that attract the general public’s attention. The existence of these conditions during pregnancy may endanger the health of the unborn baby and the mother (1–3). Pregnancy toward the second trimester induces a state of mild insulin resistance which resembles the condition imparted by inflammatory responses in non-pregnant individuals. During pregnancy, this condition results in sustained transplacental nutrient flux required for fetal growth and development (4). Compared to women with normal plasma glucose responses to carbohydrate ingestion, women with clinically significant glucose intolerance often demonstrate both increased insulin resistance and impaired insulin release and are diagnosed with gestational diabetes mellitus (GDM) (5, 6). In this respect, GDM may represent a prelude to type 2 diabetes. Indeed, a very significant number of women with GDM have been reported to develop type 2 diabetes later in life (7, 8). In pregnancy-induced diabetes, most patients are not insulin deficient, rather they have insulin resistance and high glucose levels (9). Gestational diabetes can sometimes be controlled by diet and exercise (10, 11). As a consequence of a new international detection method of diabetes as recommended by the International Association of the Diabetes and Pregnancy Study Groups (IADPSG), the incidence of GDM may be diagnosed in much higher numbers (15-18%) than currently diagnosed (5-8%) (12). Since the IADPSG method identifies increased numbers of patients with the GDM features, it has been debated whether this method is cost-effective, as compared to the current methods of diagnosis. Several studies have now concluded that the IADPSG recommendation for glucose screening during pregnancy is worthwhile and may help in preventing the onset of future diabetes (13, 14). However, the economic benefit of the IADPSG recommendation has been significantly compromised by the lack of post-delivery care. Moreover, there is a 35-40% recurrence of pregnancy-associated diabetes during a second pregnancy (15). Better screening, post-delivery counseling, and standard of care are needed to avail the benefits of the current standard of care and the IADPSG screening method. Regardless of the methods of glucose screening and the standard of clinical care, we believe improvement in the mother’s condition is due to the removal of the placenta, thus stopping the production of placental hormones resulting in severe insulin resistance. Further, we hypothesize that GDM is associated with inflammation and dysregulated immune cell activity. The placenta plays several critical roles during pregnancy: (1) transporting nutrients and waste products between mother and fetus; (2) producing and providing hormones; (3) maintaining pregnancy supportive immune environment. It is, therefore, important to determine whether pregnancy-associated diabetic conditions influence the placenta and the immune responses or vice versa.

Although GDM is a transient condition, it is a common pregnancy complication with health consequences for the mother and the fetus. It has been suggested that both the mother and the offspring are susceptible to developing chronic diseases, including obesity, diabetes, and psychological complications (16). GDM increases the risk of hypertension, fetal macrosomia, neonatal jaundice, and hypoglycemia (17). In this regard, it is critical that the contributing factors, underlying mechanisms, and therapeutic intervention strategies are identified and applied to control and treat GDM. Our novel preliminary data on proteinopathy (18), dysregulated autophagy (19, 20), and glycans-galectins (21, 22) provide insights for new mechanistic underpinnings and *in vitro and in vivo* models to better understand treatment modalities for GDM.

## Do Sir Peter Medawar’s Concepts Explain the Immunological Dysregulation in GDM?

Sir Peter Medawar proposed that an “immune tolerant” physiological state must exist during pregnancy to protect the allogeneic fetus from the mother’s immune cells (23, 24). Using the basic rules of tissue transplantation, it was proposed that a semi-allograft embryo should induce a maternal immune response that should lead to its rejection. This formed the basis for the Medawar’s “immune tolerance” or “immune privileged” hypothesis at the maternal-fetal interface (25). Since the pregnant uterus is replete with diverse immune cell types, predominately Natural Killer (NK) cells (26, 27), how is it then possible that the semi-allograft fetus is immunoprotected? It was proposed that the placenta provides a physiological barrier and that the maternal-fetal interface is an immune sterile site (28, 29). However, although the original concept of “fetal immune tolerance” is still a well-accepted phenomenon, the early theories of immune privilege and/or long-term immunosuppression at the maternal-fetal interface have been proven incorrect. If these concepts were correct, how could the *in utero* programming of spontaneous miscarriage, preterm birth, preeclampsia, and GDM be explained? We propose that an important extension of the original Medawar paradigm is the investigation into the underlying mechanisms of these adverse pregnancy outcomes. It is important to evaluate Sir Peter Medawar’s original concepts in the context of compromised metabolic pathways in the placenta, mild or acute inflammation, maternal infections, stress, starvation, michrochimerism, and diverse paternal antigen challenges.

In this review, we will focus on GDM and address the etiological issues that may disrupt immune tolerance and placental functions while not leading to fetal growth restriction, prematurity, and stillbirth. Rather, they result in excessive fetal growth, delivery complications, and post-partum health risks. These diverse effects of local immunity and inflammation in different pregnancy complications are another example of diverse triggers and pathways that impact the highly choreographed and balanced cross-talk between the maternal immune system and the placenta.

## Possible Contributory Factors to GDM

**Figure 1:**
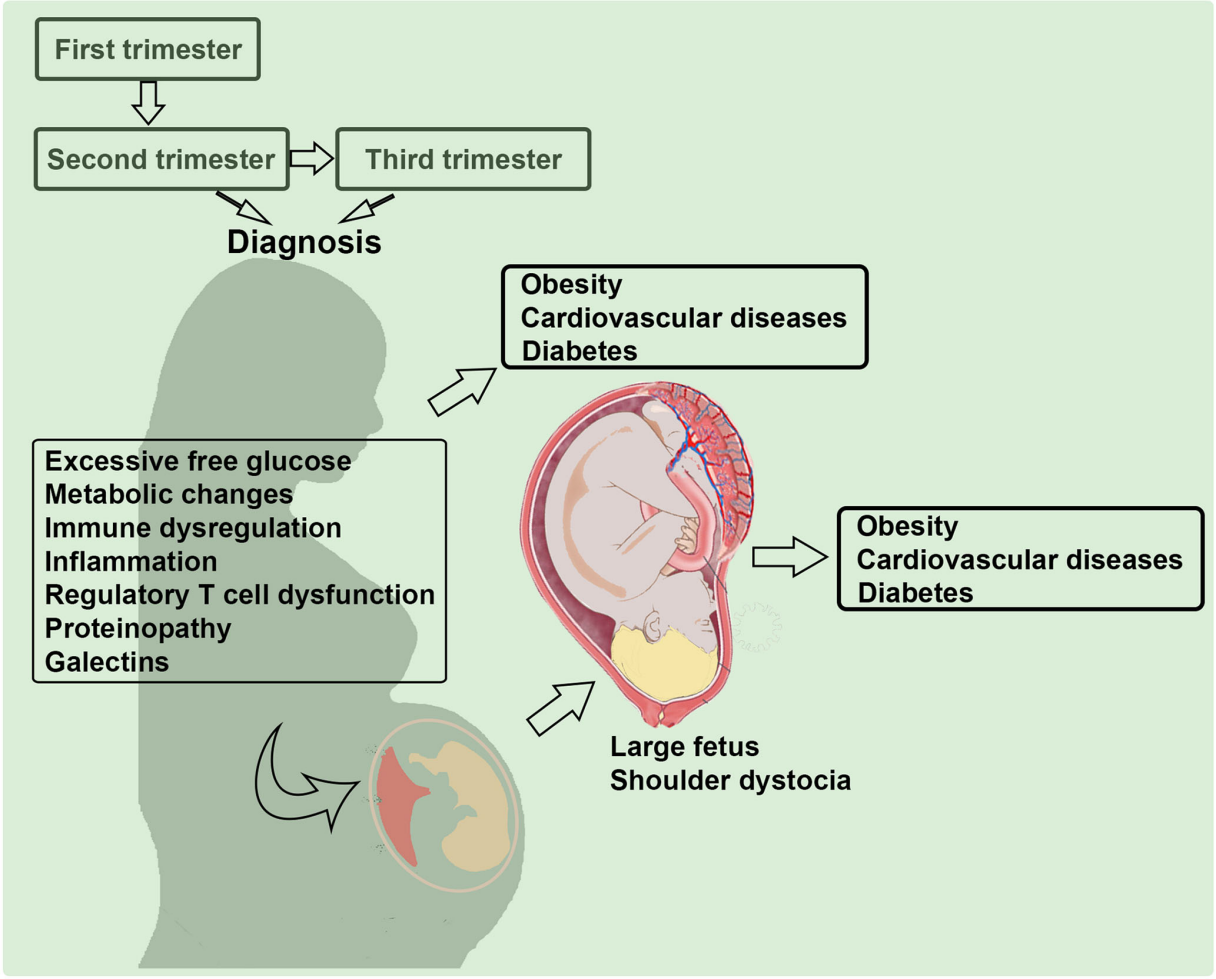
Multi-factorial etiology of gestational diabetes mellitus (GDM). Several pathways and factors that may contribute to the pathogenesis of GDM are depicted. Mild inflammation and glucose intolerance have been discussed in the literature. We propose that proteinopathy, a hallmark feature of neurodegenerative diseases, regulatory T cell dysregulation, and galectin-glycan anomalies significantly contribute to the etiology of GDM.

The etiology of GDM is possibly influenced by the multi-factorial pathways as discussed in **Figure 1**. Although the pathologic glucose intolerance in pregnant women is diagnosed at 24 weeks of gestation or later, it is imperative that the *in utero* programming begins much earlier (30, 31). Below, we discuss possible contributory factors that have attracted attention in the literature including old and new concepts such as inflammation, angiogenesis, regulatory T cell dysregulation, proteinopathy, and galectins/glycans. We will also explore immune cell dysregulation in GDM, particularly new mechanistic insights on regulatory T cells, their exhausted phenotype and include a summary of new cutting-edge concepts. Most studies highlight a nexus between excessive free glucose, gut microbiota, inflammation and dysregulated functions of immune cell types that contribute to programming of GDM (32–34). Recent birth cohort studies have implicated GDM coupled with maternal immune activation in increasing the risk of autism and schizophrenia in the offspring (35, 36). Using rodent models, GDM has also been shown to be associated with a wide range of neurodevelopmental, behavioral, and cognitive anomalies (37). The important connecting pathology here appears to be the maternal immune activation which can negatively impact placental functions, glucose tolerance, and fetal growth (38).

*a. Inflammation:* Recent evidence suggests that GDM is not only an issue of increased insulin resistance and glucose intolerance, but also a condition of low-grade systemic and placental inflammation (39). This composite pathology can lead to long term complications, including increased risk for the metabolic syndrome (obesity, insulin resistance, hypertension, dyslipidaemia and glucose intolerance) in both mother and offspring (40). Normal pregnancy has been associated with tightly regulated inflammatory reactions that are critical to implantation and the process of spontaneous labor. To sustain fetal-placental growth, the mother increases glucose intake, becomes partially glucose intolerant, and exhibits insulin resistance, which results in the body shifting from lipid storage to lipolysis in order to achieve enough energy to sustain normal metabolism (41). In GDM, maternal immune tolerance as well as placental balance of inflammation and anti-inflammation is disrupted and may program excessive insulin resistance (42). Recent findings further suggest that both placental and visceral adipose tissue play critical part in instigating and mediating this low-grade inflammatory response which involves altered infiltration, differentiation and activation of maternal innate and adaptive immune cells. Adipose tissue is a complicated organ made up of several different cell types that have different energy storage, metabolic control, and immunological activities (43). It includes a variety of immune cells, both adaptive immune cells (B and T lymphocytes; regulatory T cells) and innate immune cells (primarily macrophages and myeloid-derived suppressor cells) (44). At the crossroads of metabolism and immunity, adipose tissue is increasingly recognized as a legitimate immune organ. It secretes IL-1, IL-6, IL-8, IL-10, TNF-α, and monocyte chemoattractant protein-1 (MCP1) (43). MCP1 also affects insulin sensitivity, increases macrophage recruitment, and contributes to inflammation (45). When the balance is skewed toward the production of inflammatory effectors such as leptin, TNF-α, and IL-6 with reduced production of adiponectin, it may lead to insulin resistance and the onset of diabetic condition (46). Inflammation caused by secreted inflammatory cytokines is believed to be linked to increased insulin resistance in GDM (47). It is believed that adipocytes release pro-inflammatory cytokines that act on local immune cells to induce further excessive production of pro-inflammatory cytokines, culminating in local and systemic inflammation (48). This may also affect systemic and local immune cell numbers and functions. Based on these observations, the management of GDM may also be achieved by targeting normalization of inflammation and immune cell profiles and functions.

*b. Adaptive immunity and regulatory T cells in GDM:* Type 2 diabetes and GDM entail similar diagnostic and etiological features. In type 2 diabetes, both innate and adaptive immunity responses have been shown to play a role in maintaining low grade inflammation and immune cytotoxic environment (49, 50). Similarly, in GDM, the hyperglycemic condition generates a proinflammatory environment capable of changing the phenotype of NK cells and cytokines in the maternal circulation and the placental-fetal unit (51). Hara et al. analyzed peripheral blood NK cells and their counterparts in the decidua and reported that increased presence of both cytotoxic and cytokine-secreting NK subsets was observed at the maternal-fetal interface in GDM patients as compared to women undergoing a normal pregnancy (52). Similar observations were made by Chiba et al. in that the proportion of IFNγ- and TNF-α-producing CD56^+^ NK cells was increased, whereas the number of TGF-β- and VEGF-producing CD56^+^ NK cells decreased in GDM patients. These findings suggest that GDM patients have an elevated number of cytotoxic NK cells (53, 54). In normal pregnancy, the decidua contains macrophages of predominantly the M2 subtype (anti-inflammatory). In contrast, recent studies reported that M2 macrophages switch to M1 macrophages of pro-inflammatory phenotype in GDM patients (55, 56). Although these innate immune responses have been documented in GDM patients, literature on NK cells and macrophages is still debatable. For example, in GDM patients NK cells and NKCD56^bright^/NKp46+ were shown to be higher than that in controls, suggesting GDM patients have an increased number of non-cytotoxic NK cells (57). Uterine CD56^bright^CD16^dim^ NK cells are hallmark of normal pregnancy and present in very high numbers in the decidua during early pregnancy. However, it is not clear why CD56^bright^ NK cell subpopulation increases in GDM. Likewise, the influence of GDM on the quantity and function of placental macrophages is a subject of debate. Some studies reported that in pregnancies with infection and diabetes, placental Hoffbauer cells (mostly M2) appear to be converted to a pro-inflammatory M1 phenotype (57). It is still not known what happens to decidual or adipocyte macrophages. Thus, the main emphasis continues to be placed on dysregulation of CD4^+^ T cells, particularly CD4^+^CD25^+^FoxP3^+^ regulatory T cells (Tregs) and their modulation by checkpoint molecules or propagation of Th17 cells, as these cells are also embedded in adipose tissue and contribute to control of inflammation and mild autoimmune reactions (58).

Regulatory T cells (Tregs) were first identified in rodents as thymus-derived naturally occurring suppressive CD4^+^CD25^+^ T cells actively controlling the maintenance of peripheral self-tolerance as their depletion led to spontaneous development of several types of autoimmune conditions similar to those diagnosed in humans (59, 60). Their role in controlling autoimmunity was further confirmed by prevention of such conditions by reconstituted Tregs (61, 62). These Tregs were later found to uniquely express the transcription factor forkhead box protein 3 (FoxP3) (63, 64). The research over the last three decades or so suggest that Tregs control a variety of pathological and physiological immune responses, including tumor immunity, autoimmunity, microbial immunity, and most importantly for this discussion fetal immune tolerance (65, 66). GDM is an ideal clinical setting where Tregs can be targeted to suppress low-grade inflammation and adverse immune responses.

Recent research has focused on the mechanisms that contribute to dysregulated functions of Tregs. Since thymic Tregs are antigen responsive as recognized by intrinsic CD25 and CTLA-4 expression, they can be easily activated and can recognize self-antigens in the periphery to maintain immune tolerance (67). Are these cells functionally compromised in GDM is a topic of considerable debate. Although subtypes of Tregs have been shown to be diminished in numbers, preliminary results from our lab support the notion that peripheral Tregs numbers remain almost the same in normal pregnancy and GDM pregnancies (68). Both peripheral and decidual Tregs increase during pregnancy and play an important role in implantation; however, it is not clear what role they play beyond implantation as their depletion in pregnant mice on gestational day 7 or beyond does not seem to affect pregnancy outcome. We propose that in GDM, Tregs either lose their immunosuppressive functions or acquire an inflammatory phenotype by virtue of producing inflammatory cytokines. In type 2 diabetes patients, decreased Treg numbers have been observed and this has been associated with high glucose and high-density lipoproteins in blood (69). We propose that elevated expression of immune checkpoint molecules on Tregs, such as PD-1, as a result of epigenetic modifications imparts non-functional phenotype of these cells in GDM. It is quite possible that disrupted metabolic pathways in GDM may induce epigenetic changes or inflammatory phenotype in Tregs. A recent study provides a novel link between the receptor activator of NF-kB ligand (RANK) and the onset of GDM (70). It is proposed that the pregnancy hormone progesterone drives expansion of natural Tregs through RANK and its thymic deletion may result in impaired accumulation of Tregs in visceral adipose tissue. This accumulation may be associated with enlarged adipocyte size, tissue inflammation, enhanced maternal glucose intolerance, fetal macrosomia, and long-lasting alteration in glucose homeostasis, all key features of GDM. This study also suggests that reduced RANK expression in GDM is associated with reduced number of Tregs in the human placenta. Other reports suggest that in third trimester of pregnancy GDM patients had a higher proportion of Tregs compared to normal pregnancy controls. It is quite possible that Treg numbers may fluctuate in a trimester-dependent manner and be accordingly affected by metabolic anomalies. Accordingly, it has recently been shown that the content of a newly identified immunosuppressive cytokine IL-35 which is exclusively produced by Treg cells is decreased in GDM patients (71). Taken together, these observations clearly point to an important role of Tregs in GDM. Further investigation is warranted to delineate any correlation between Treg number/proportion and their functional phenotype such as immune checkpoint molecule expression.

Recent investigations also suggest that there is expansion of Th17 cells in GDM. These cells express the transcription factor RORγT and produce IL-17, a cytokine involved in inducing inflammation and recruitment of neutrophils. In normal pregnancy, Th17 cells are not present at the maternal-fetal interface in any significant numbers (58). These observations raise the question whether these CD4^+^ T cells increase in response to metabolic changes and recruited to the maternal-fetal interface or whether they are differentiated from Tregs due to local inflammation. In addition, a question can also be posed whether peripheral Th17 cells contribute to the systemic symptoms in GDM. It is known that IL-6 is an upstream regulator of IL-17 (72, 73), and recent studies suggest distinct elevated production of IL-6 in GDM patients. It is then possible that there is an excessive peripheral and decidual presence of Th17 cells in pregnant women with metabolic syndromes. Th17 cells are pro-inflammatory and enhance the phagocytic or cytotoxic activity of macrophages and neutrophils by secreting IL-17. These observations suggest that a threshold ratio of Tregs and Th17 may be a critical parameter to gauge the onset of GDM-like pathology.

*c. Galactins-Glycans and GDM:* Galectins consist of an endogenous family of β-galactoside-binding animal proteins defined by a conserved carbohydrate recognition domain (CRDs) of approximately 130 amino acids (74). This glycan binding proteins family of 15 members exert regulatory functions at the feto-maternal interface with implications in implantation, trophoblast development, placentation, maternal immune and vascular adaptations (22). Based on their structure, galectins have been classified in three groups: the proto-, chimeric- and tandem-repeat type. Prototype galectins contain only one CRD that may self-associate to form homodimers (e.g. gal-1, -2, -13 among others). Tandem-repeat galectins consist of two distinct CRDs linked by up to 70 amino acids (e.g. gal-9). Finally, gal-3, the only chimeric type galectin, is characterized by a single CRD linked to a N terminal domain. Galectins act as endogenous decoders translating glycan-containing information into an extensive spectrum of cellular responses including immune and vascular signaling programs *via* receptor clustering, reorganization and endocytosis (74). Metabolism and inflammation are fine-tuned events regulated by members of the galectin family. For instance, gal-1 depicts a critical role during the initiation of the adaptive response by limiting the capability of antigen presenting cells and inducing apoptosis in activated T cells (Th1/Th17). The high gal-1 expression on the Treg subset further modulates the adaptive response upon immune activation (74). Other members, e.g. gal-3, interacts with chemokines involved in the late stage of inflammation which may attenuate or resolves inflammation (75). Placental specific galectin (gal-13) induced apoptosis of T cells conferring additional immune tolerance mechanisms to sustain the semi allograft fetus (76).

**Figure 2:**
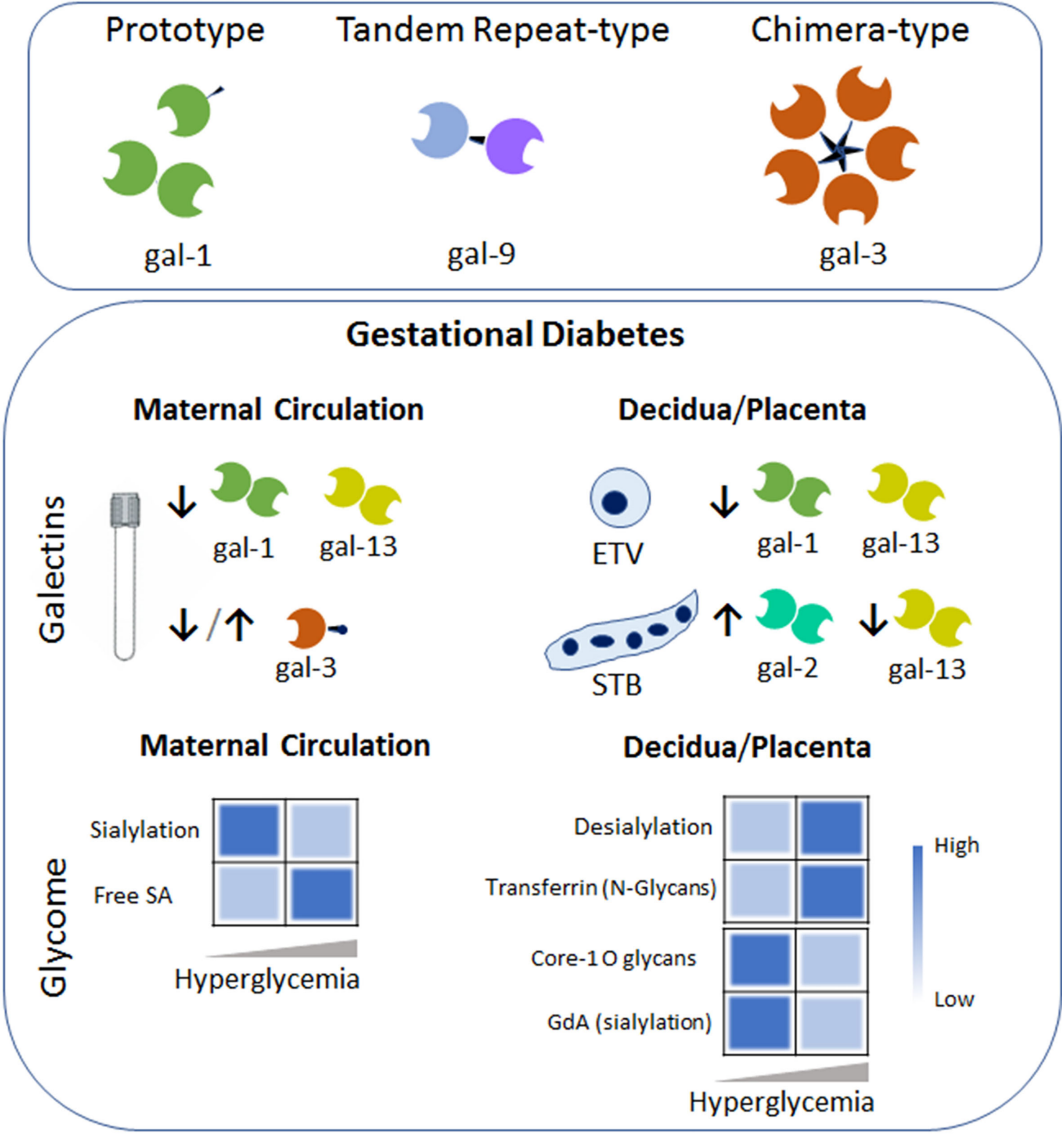
Galectins-Glycans circuits in GDM. The galectin family members are divided into three types: the prototype (e.g. galectin-1 (gal-1)) with one carbohydrate recognition domain (CRD), the tandem–repeat type (gal-9) with two CRDs connected by a non-conserved linker and the chimeric type with one CRD and a non-lectin N-terminal domain (gal-3). Some galectins can self-associate into dimers or oligomers. During GDM an aberrant galectin signature characterizes the maternal circulation and the placental niche (e.g. within the extravillous trophoblast (ETV) and Syncytiotrophoblast (STB). Arrows denoted up-regulated (↑) or down-regulated (↓) expression compared to the uneventful gestation. Heatmap based on the relative levels expression of the glycome in the maternal circulation (left) and placenta compartment (right) during the course of GDM. Changes in glycosylated protein composition (e.g. Transferrin or Glycodelin-A (GdA)) in gestational diabetes are summarized. Dysregulation of sialic acid (SA) dynamics might contribute to the pro-inflammatory milieu in maternal circulation and placental dysfunction.

Several galectin members (e.g. gal-1, -3, -9 and -13) gradually increase their concentrations in the bloodstream as normal pregnancy progressed (77–79). However, women with pregnancy complications specifically those suffering from GDM failed to upregulate gal-1 concentration during gestation (21). Moreover, Unverdorben L. and colleagues showed a systemic decrease in another prototype galectin member (gal-13) in women diagnosed with GDM (80). A failure to up-regulate gal-3 concentrations in the bloodstream after the onset of GDM has been described (78). However, two different investigations have shown increased circulation of gal-3 in women diagnosed with GDM (69, 81). In addition, the Talmor-Barkan Y et al. study also showed elevated gal-3 levels in the bloodstream during the first trimester in women who subsequently developed GDM (81). Furthermore, neonates from GDM mothers have higher gal-3 level in cord blood when compared to those from uneventful pregnancies (82). Studies investigating the chimera type galectin mainly differ in the population included in the clinical cohort used (especially in the BMI and ethnicity of the GDM group) and also in the pairs of antibodies used in the ELISA determination. Therefore, the augmentation of gal-3 systemic levels observed in women suffering from GDM could be related to the higher BMI of these patients rather that the pregnancy complication itself. Nevertheless, galectins have been already implicated in several metabolic diseases including diabetes, obesity, and atherosclerosis. For instance, it has been shown that gal-3 causes insulin resistance in certain stages of diabetes reducing the glucose tolerance and insulin sensitivity in muscle cells, hepatocytes, and adipose cells. Therefore, the increased levels of gal-3 and other placental hormones in the bloodstream caused cellular and systemic insulin dysfunction during gestation. Actions of circulating galectins in the bloodstream of pregnant women are not yet fully understood and represent a metabolic target to prevent or reverse gestational diabetes (**Figure 2**).

Placenta homeostasis requires an adequate and balanced trophoblast metabolism to ensure a proper fetal development. However, metabolic disorders such as gestational diabetes cause complications. Over the past decade, a distinguished trophoblast galectin fingerprint was found to be associated with this metabolic disorder (**Figure 2**). For instance, we were able to show that GDM is characterized for a decreased gal-1 expression in extra-villous trophoblast cells (ETV) within the maternal decidua (21). Moreover, placental gal-1 secretion was less sensitive to high glucose concentration compared to low concentration glucose, demonstrating the inverse correlation between glucose and gal-1 found in GDM patients. We also proposed that the inverse correlation between glucose and gal-1 is associated with the maternal exacerbated immune response (e.g. TNF-alpha, IL-6 and adipocytokines), which might aggravate the metabolic disorder. Interestingly, expression of gal-2 is increased in the syncytiotrophoblast (STB) layer of the placentas from GDM patients compared to uneventful gestations (83). Although sex-specific differences are common in placental disorders, the increase of gal-2 expression during the gestational diabetes was not influenced by the gender of the fetuses. At the maternal decidua, the majority of gal-2 expressing cells were identified as ETV in women who suffered from GDM. Association between *LGALS1* (gal-1 gene) and GDM complicated pregnancy (21), *LGALS2* (gal-2 gene) and fasting insulin and glucose has been reported (84), however, further studies are necessary to understand their roles in the development of metabolic disorder during gestation. In addition, gal-13 expression in STB and ETV/decidua was reported to be downregulated in term GDM placentas (80). However, when placentas derived from GDM patients were analyzed, gal-3 was not found to be dysregulated compared to uneventful gestations (78). Together these studies indicate that the galectin fingerprint is likely related metabolic complication during gestation through multiple mechanisms including the regulation of pro-inflammatory cytokines and placenta function.

Women with GDM are at risk of pathological outcome associated with impaired immune regulation and abnormal carbohydrate metabolism, leading to alteration of gene expression and activities of the cellular glycosyltranferases and glycosidases. In addition, the dynamic of sialic acid (SA) determines the function of cells and is highly associated with human health and disease. The sialylation is regulated by sialyltransferases which installs the SA group, whereas desialylation (sialidases) removes it. During diabetes, the sialyltransferase activity is reduced and an increased free sialic acid level in serum has been observed. In addition, placental sialidase activity is increased in patients with GDM (85, 86). Moreover, challenge of JEG-3 cells (a choriocarcinoma cell line) with high glucose (25mM) medium significantly decreased the α2,3-sialylation (determined by Maackia amurensis lectin; MAA) and core 1 O-glycans (identified by Arachis hypogaea lectin; PNA) as compared to JEG-3 cells maintained in low glucose (5.5mM) medium (**Figure 2**). Changes in the glycosylation process of proteins in GDM influences the immunomodulatory function of glycoproteins during pregnancy. For instance, an increase in N-glycosylation is associated with decreased transferrin binding capacity of transferrin placenta receptor (87). Differentially glycosylated placenta proteins include human chorionic gonadotropin (88). In addition, hyperglycemia causes the production of advanced glycation end products, leading to glycomodification of decidual proteins, e.g. glycodelin-A (GdA), an abundant secretory glycoprotein of the decidua. Although changes in glycosylation of placental and decidual proteins occurs during normal pregnancy, reduction of α2,6 sialylation of GdA during GDM provokes a reduced apoptosis-inducing activity on lymphocytes, thus decreasing GdA immunomodulatory function (89). Taking these findings into account, we hypothesize that alteration in the placental/decidual carbohydrate composition during GDM development results in impaired binding activities including the galectin-glycan interactions. We further propose that alteration in placental glycan composition could potentially be useful as a biomarker in cases of GDM.

*d. Proteinopathy:* There have been suggestions in the literature that patients with chronic type 2 diabetes are at high risk of developing Alzheimer’s disease (AD) (90, 91). A hallmark diagnostic feature of AD is defined by seeded growth and histopathological evidence of extracellular amyloid β (Aβ) plaques and intracellular neurofibrillary tangles involving diverse hyperphosphorylated tau isoforms in the post-mortem brain (92, 93). The process of protein aggregation is termed proteinopathy. It is unknown whether proteinopathy is also induced in response to the metabolic syndrome conditions such as GDM. We have recently developed a novel blood test to detect protein aggregates in serum samples from preeclampsia, GDM, and AD patients (18). Our preliminary results suggest that there is evidence of proteinopathy in GDM; however, protein aggregate components differ among these conditions, suggesting that these pathologies share a common mechanistic pathway, albeit with diverse protein aggregate complexes. This is a novel finding and warrants a thorough study not only in GDM but other conditions.

## Animal Models of GDM That Mimic the Features of the Human Condition

Several mouse and rat models have been developed to better understand the pathophysiology of GDM (94–96). These models further highlight the maternal and fetal outcomes resembling the human condition induced by multiple factors, including nutritional, pharmacological, and stress as well as placental signaling and fetal outgrowth. Interestingly, a mouse model has been developed to study the maternal immune activation and its effects in the developing brain (97). This model suggests that the GDM-like condition and the maternal immune activation resulted in altered inflammatory and neurodevelopmental transcriptome profiles. One caveat with several of these models is the use of high fat diet during the pre-pregnancy period and throughout the pregnancy period (98). This approach could program inflammatory conditions with pregnancy-incompatible visceral fat and cytokines. In addition, both systemic and uterine immune profiles could be skewed toward inflammatory milieu that can induce insulin resistance with or without pregnancy. Nevertheless, these models provide important insights in understanding the pathophysiology of GDM-like metabolic syndromes.

**Figure 3:**
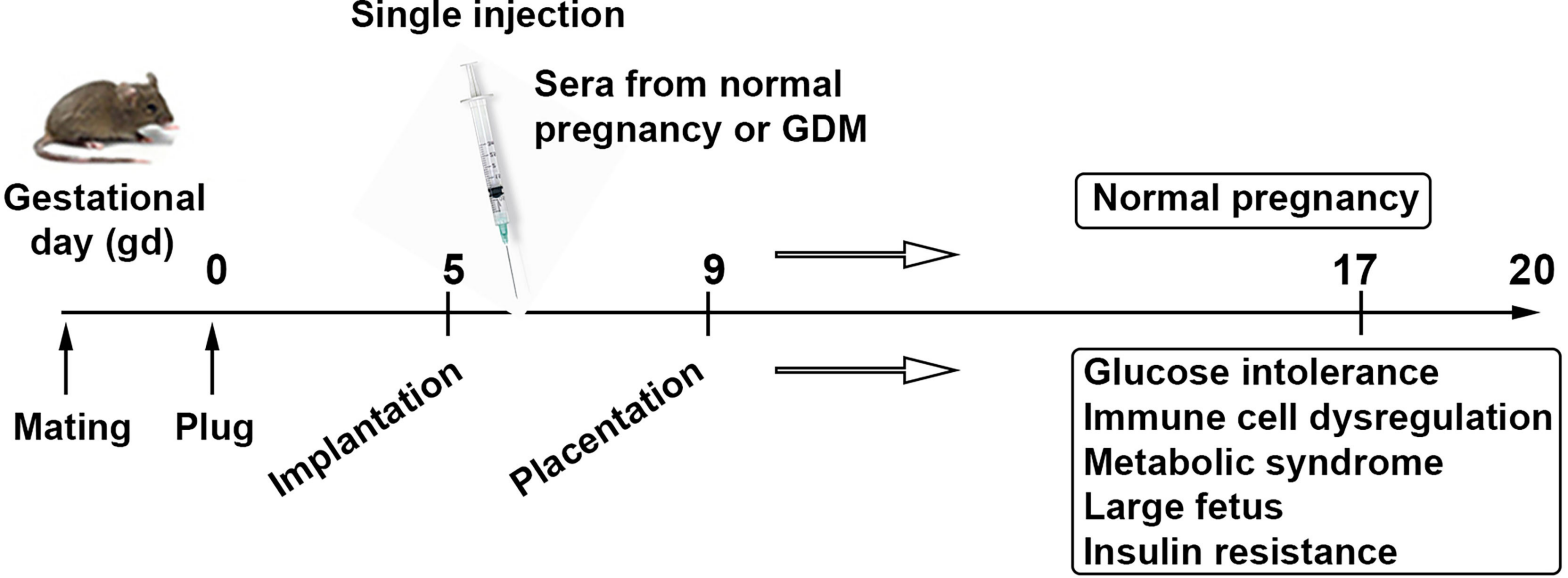
Serum-based proposed animal model of GDM. Schematic presentation describes a strategy for a humanized mouse model of gestational diabetes mellitus mimicking metabolic and immune pathologies of the human pregnancy complication. It is a human serum-based model of GDM. Analysis of serum, placental tissue, fetal weight and size, placental insulin resistance signaling, and peripheral and uterine immune profiles is expected to recapitulate all the features associated with GDM.

We have recently developed a semi-humanized mouse model of GDM by injecting serum from normal pregnancy controls and GDM patients. Our model requires a single serum injection at a defined gestational age, and serum from GDM, not normal pregnancy, induces all the features of GDM in humans. As shown in **Figure 3**, the fetal growth is excessive in units from mice that were administered serum from GDM patients. These mice also exhibit glucose intolerance, Treg dysregulation, insulin resistance in the placenta, and proteinopathy. Our model does not require any pre-pregnancy feeding of high fat diet, suggesting that serum from GDM patients contains key factors that can cause GDM-like features in pregnant mice.

## Conclusions

We have presented a comprehensive overview of the literature on GDM and proposed new ideas with discussion of our experimental strategy to better understand the GDM etiology. We highlight the fact that the maternal-fetal tolerance choreography is not a uniform event in normal pregnancy vs. adverse pregnancy outcomes such as GDM. The mechanisms that dysregulate the maternal immune system, placental metabolic pathways, fetal growth, and fetal neurodevelopment in GDM are vastly different than those in normal pregnancy and even those proposed for normal pregnancy by Sir Peter Medawar and should be further studied. We emphasize the importance of low-grade inflammation, dysregulated Tregs and Th17 cells, galectins-glycans signaling pathways, and proteinopathy in the programming of GDM (see **Figure 1**). We have referred to some published information on mouse and rat models of GDM and briefly described a humanized mouse model of this common pregnancy complication with health risk for both mother and offspring later in life. We believe that important insights to better understand the pathophysiology of GDM can be derived from well-defined animal models.

## Author Contributions

SS and SMB contributed to conceptualization. All authors participated in writing, correcting, and editing the manuscript. All authors contributed to the article and approved the submitted version.

## Funding

This work was supported in part by the NIH P20 GM121298, 3P20GM121298-04W1 and P30 GM114750 grants, Brown University DEANS Award, and Brown University Seed Award to SS. SMB was supported by the Deutsche Forschungsgemeinschaft (DFG) through the Heisenberg Program (BL1115/7-1) and research grants from the DFG (BL1115/2-1, BL1115/4-1) and the Heike und Wolfgang Mühlbauer Stiftung.

## Conflict of Interest

The authors declare that the research was conducted in the absence of any commercial or financial relationships that could be construed as a potential conflict of interest.

## Publisher’s Note

All claims expressed in this article are solely those of the authors and do not necessarily represent those of their affiliated organizations, or those of the publisher, the editors and the reviewers. Any product that may be evaluated in this article, or claim that may be made by its manufacturer, is not guaranteed or endorsed by the publisher.
